# Supplementary material for: Multimorbidity Patterns and Their Association with Social Determinants, Mental and Physical Health during the COVID-19 Pandemic
Source: Int J Environ Res Public Health. 2022 Dec 15;19(24):16839. doi: 10.3390/ijerph192416839 (PMC9778742; doi:10.3390/ijerph192416839)
Supplement: Supplementary file 1 [file ijerph-19-16839-s001.zip › ijerph-2035066-supplementary.pdf]

**Tables:**

Table S1: Summary of the sample characteristics by Covid-19 (n = 1592)

Table S2: Factorial structure of the SF-12 scale

Table S3: Summary of the prevalence of chronic conditions in each class (n = 1592)

Table S4: Summary of the sample characteristics by multimorbidity pattern (n = 1592)

Figure S1: COVID-19 by Multimorbidity Pattern

Figure S2: Sequelae COVID-19 by Multimorbidity Pattern

**Table S1:** Summary of the sample characteristics by Covid-19 (n = 1592)

| Characteristics | Overall | Covid-19<br>N = 302 | No Covid-19<br>N = 1290 |
|---|---|---|---|
| Multimorbidity | 4.77 (2.78) | 4.78 (2.89) | 4.67 (2.77) |
| Gender | | | |
| Male | 678 (43%) | 130 (43%) | 548 (42%) |
| Female | 914 (57%) | 172 (57%) | 742 (58%) |
| Age | | | |
| 50-59 | 543 (34%) | 140 (46%) | 403 (31%) |
| 60-69 | 481 (30%) | 96 (32%) | 385 (30%) |
| >69 | 568 (36%) | 66 (22%) | 502 (39%) |
| Disability | | | |
| Yes | 282 (18%) | 54 (18%) | 228 (18%) |
| No | 1,307 (82%) | 247 (82%) | 1,060 (82%) |
| Education | | | |
| No | 206 (13%) | 30 (9.9%) | 176 (14%) |
| Primary | 625 (39%) | 124 (41%) | 501 (39%) |
| Secondary | 468 (29%) | 95 (31%) | 373 (29%) |
| University | 291 (18%) | 53 (18%) | 238 (18%) |
| Fruits and Vegetables | | | |
| One per week or less | 145 (9.1%) | 33 (11%) | 112 (8.7%) |
| 4 times per week | 469 (29%) | 91 (30%) | 378 (29%) |
| One per day | 422 (27%) | 74 (25%) | 348 (27%) |
| Two or more per day | 556 (35%) | 104 (34%) | 452 (35%) |
| Physical Activity | | | |
| Never | 479 (30%) | 93 (31%) | 386 (30%) |
| One per month or less | 97 (6.1%) | 20 (6.6%) | 77 (6.0%) |
| Several times per week | 508 (32%) | 105 (35%) | 403 (31%) |
| All days | 508 (32%) | 84 (28%) | 424 (33%) |
| Alcohol Consumption | | | |
| Never | 648 (41%) | 116 (38%) | 532 (41%) |
| One per month or less | 106 (6.7%) | 20 (6.6%) | 86 (6.7%) |
| One per week or less | 339 (21%) | 85 (28%) | 254 (20%) |
| Several times per week | 499 (31%) | 81 (27%) | 418 (32%) |
| Tobacco Consumption | | | |
| Neither smokes nor has smoked | 701 (44%) | 123 (41%) | 578 (45%) |

| | | | |
|---|---|---|---|
| Used to smoke | 631 (40%) | 142 (47%) | 489 (38%) |
| 1-10 cigarettes | 149 (9.4%) | 22 (7.3%) | 127 (9.8%) |
| >10 cigarettes | 111 (7.0%) | 15 (5.0%) | 96 (7.4%) |
| Job Situation | | | |
| Active | 403 (25%) | 106 (35%) | 297 (23%) |
| Retiree | 730 (46%) | 112 (37%) | 618 (48%) |
| Unemployed | 144 (9.0%) | 36 (12%) | 108 (8.4%) |
| Domestic Work | 309 (19%) | 47 (16%) | 262 (20%) |
| Income | | | |
| <600€ | 122 (7.7%) | 28 (9.3%) | 94 (7.3%) |
| 601€-900€ | 309 (19%) | 44 (15%) | 265 (21%) |
| 901€-1200€ | 343 (22%) | 73 (24%) | 270 (21%) |
| 1201€-1800€ | 334 (21%) | 69 (23%) | 265 (21%) |
| >1800€ | 300 (19%) | 62 (21%) | 238 (18%) |
| No answer | 184 (12%) | 26 (8.6%) | 158 (12%) |
| SF12 (Physical) | 39.18 (6.44) | 38.95 (6.41) | 39.80 (5.90) |
| SF12 (Mental) | 43.34 (6.95) | 43.31 (6.96) | 43.82 (6.13) |
| Administrative Region | | | |
| Cadiz Bay (Ref) | 196 (12%) | 37 (12%) | 159 (12%) |
| Jerez and Rural | 631 (40%) | 123 (41%) | 508 (39%) |
| Gibraltar Zone | 190 (12%) | 39 (13%) | 151 (12%) |
| Northwest Coast | 199 (12%) | 34 (11%) | 165 (13%) |
| La Janda | 180 (11%) | 33 (11%) | 147 (11%) |
| Cadiz Mountains | 196 (12%) | 36 (12%) | 160 (12%) |
| Primary Attention Visit | | | |
| Yes | 1,239 (78%) | 235 (78%) | 1,004 (78%) |
| No | 353 (22%) | 67 (22%) | 286 (22%) |
| Emergencies Visit | | | |
| Yes | 535 (34%) | 103 (34%) | 432 (33%) |
| No | 1,057 (66%) | 199 (66%) | 858 (67%) |
| Hospital Admission | | | |
| Yes | 170 (11%) | 30 (9.9%) | 140 (11%) |
| No | 1,422 (89%) | 272 (90%) | 1,150 (89%) |
| Specialist Visit | | | |
| Yes | 808 (51%) | 145 (48%) | 663 (51%) |
| No | 784 (49%) | 157 (52%) | 627 (49%) |

**Table S2:** Factorial Structure of the SF-12 Scale

| Questions | Subscale of SF-36 | D1 (PCS) | D2 (MCS) |
|---|---|---|---|
| PF02 | Physical Functioning | **0.77** | 0.19 |
| PF03 | | **0.72** | 0.18 |
| RP2 | Role Physical | **0.73** | 0.20 |
| RP3 | | **0.73** | 0.24 |
| BP2 | Bodily Pain | **-0.73** | -0.29 |
| GH1 | General Health | **-0.55** | -0.28 |
| VT2 | Vitality | **-0.48** | **-0.42** |
| SF2 | Social Functioning | **0.45** | **0.48** |
| RE2 | Role Emotional | 0.14 | **0.82** |
| RE3 | | 0.18 | **0.77** |
| MH3 | Mental Health | -0.24 | **-0.47** |
| MH4 | | 0.28 | **0.66** |
| Eigenvalues | | 3.63 | 2.66 |
| Variance Explained | | 32.25 | 25.42 |

**Table S3:** Summary of the prevalence of chronic conditions in each class (n = 1592)

| Characteristics | Overall N = 1592 | C1 N = 448 | C2 N = 659 | C3 N = 247 | C4 N = 76 | C5 N = 162 |
|---|---|---|---|---|---|---|
| High blood pressure | 792 (50%) | 56 (12%) | 485 (74%) | 99 (40%) | 26 (34%) | 126 (78%) |
| Heart attack | 70 (4.4%) | 2 (0.4%) | 43 (6.5%) | 1 (0.4%) | 1 (1.3%) | 23 (14%) |
| Angina | 56 (3.5%) | 0 (0%) | 33 (5.0%) | 0 (0%) | 1 (1.3%) | 22 (14%) |
| Other heart problems | 171 (11%) | 13 (2.9%) | 78 (12%) | 8 (3.2%) | 3 (3.9%) | 69 (43%) |
| Varicose veins | 324 (20%) | 67 (15%) | 96 (15%) | 63 (26%) | 37 (49%) | 61 (38%) |
| Arthrosis | 590 (37%) | 119 (27%) | 144 (22%) | 148 (60%) | 45 (59%) | 134 (83%) |
| Cervical back pain | 515 (32%) | 69 (15%) | 42 (6.4%) | 247 (100%) | 52 (68%) | 105 (65%) |
| Lumbar back pain | 578 (36%) | 89 (20%) | 82 (12%) | 245 (99%) | 59 (78%) | 103 (64%) |
| Chronic allergy or dermatitis | 347 (22%) | 143 (32%) | 61 (9.3%) | 62 (25%) | 23 (30%) | 58 (36%) |
| Asthma | 108 (6.8%) | 32 (7.1%) | 21 (3.2%) | 19 (7.7%) | 5 (6.6%) | 31 (19%) |
| EPOC | 113 (7.1%) | 20 (4.5%) | 41 (6.2%) | 10 (4.0%) | 5 (6.6%) | 37 (23%) |
| Diabetes | 299 (19%) | 3 (0.7%) | 189 (29%) | 26 (11%) | 2 (2.6%) | 79 (49%) |
| Stomach ulcer | 75 (4.7%) | 35 (7.8%) | 10 (1.5%) | 17 (6.9%) | 5 (6.6%) | 8 (4.9%) |
| Urinary incontinence | 145 (9.1%) | 19 (4.2%) | 44 (6.7%) | 21 (8.5%) | 20 (26%) | 41 (25%) |
| High cholesterol | 608 (38%) | 138 (31%) | 252 (38%) | 85 (34%) | 15 (20%) | 118 (73%) |
| Cataracts | 278 (17%) | 36 (8.0%) | 118 (18%) | 30 (12%) | 11 (14%) | 83 (51%) |
| Chronic skin problems | 154 (9.7%) | 57 (13%) | 39 (5.9%) | 27 (11%) | 14 (18%) | 17 (10%) |
| Chronic constipation | 117 (7.3%) | 33 (7.4%) | 6 (0.9%) | 29 (12%) | 24 (32%) | 25 (15%) |
| Depression | 215 (14%) | 37 (8.3%) | 23 (3.5%) | 19 (7.7%) | 75 (99%) | 61 (38%) |
| Anxiety | 219 (14%) | 50 (11%) | 24 (3.6%) | 27 (11%) | 69 (91%) | 49 (30%) |
| Ictus | 46 (2.9%) | 4 (0.9%) | 17 (2.6%) | 3 (1.2%) | 2 (2.6%) | 20 (12%) |
| Chronic headache | 209 (13%) | 84 (19%) | 10 (1.5%) | 46 (19%) | 33 (43%) | 36 (22%) |
| Hemorrhoids | 213 (13%) | 73 (16%) | 41 (6.2%) | 35 (14%) | 21 (28%) | 43 (27%) |
| Malignant tumor | 88 (5.5%) | 45 (10%) | 17 (2.6%) | 8 (3.2%) | 2 (2.6%) | 16 (9.9%) |
| Ostheoporosis | 132 (8.3%) | 62 (14%) | 4 (0.6%) | 18 (7.3%) | 14 (18%) | 34 (21%) |
| Thyroid problem | 208 (13%) | 80 (18%) | 35 (5.3%) | 43 (17%) | 6 (7.9%) | 44 (27%) |
| Kidney problem | 93 (5.8%) | 27 (6.0%) | 23 (3.5%) | 9 (3.6%) | 8 (11%) | 26 (16%) |
| Prostate problem | 141 (8.9%) | 43 (9.6%) | 50 (7.6%) | 18 (7.3%) | 7 (9.2%) | 23 (14%) |
| Menopause problem | 82 (5.2%) | 51 (11%) | 6 (0.9%) | 10 (4.0%) | 9 (12%) | 6 (3.7%) |
| Accident injury | 128 (8.0%) | 37 (8.3%) | 37 (5.6%) | 18 (7.3%) | 21 (28%) | 15 (9.3%) |
| Obesity | 438 (28%) | 54 (12%) | 234 (36%) | 67 (27%) | 25 (33%) | 58 (36%) |

**Table S4:** Summary of the sample characteristics by multimorbidity pattern (n = 1592)

| Characteristics | C1, N = 448 (28%) | C2, N = 659 (41%) | C3, N = 247 (16%) | C4, N = 76 (5%) | C5, N = 162 (10%) |
|---|---|---|---|---|---|
| Multimorbidity | 3.47 (1.52) | 3.51 (1.51) | 5.94 (2.05) | 8.49 (2.64) | 9.80 (2.46) |
| SF12 (Physical) | 40.49 (5.67) | 39.57 (5.96) | 38.21 (6.83) | 39.30 (7.48) | 35.32 (7.45) |
| SF12 (Mental) | 43.84 (6.62) | 44.21 (6.19) | 43.18 (7.23) | 38.28 (8.42) | 40.96 (8.02) |
| Administrative Region | | | | | |
| Cadiz Bay | 64 (14%) | 78 (12%) | 30 (12%) | 9 (12%) | 15 (9.3%) |
| Jerez and Rural | 174 (39%) | 269 (41%) | 88 (36%) | 28 (37%) | 72 (44%) |
| Gibraltar Zone | 60 (13%) | 73 (11%) | 31 (13%) | 11 (14%) | 15 (9.3%) |
| Northwest Coast | 56 (12%) | 73 (11%) | 38 (15%) | 9 (12%) | 23 (14%) |
| La Janda | 42 (9.4%) | 87 (13%) | 26 (11%) | 9 (12%) | 16 (9.9%) |
| Cadiz Mountains | 52 (12%) | 79 (12%) | 34 (14%) | 10 (13%) | 21 (13%) |
| Gender | | | | | |
| Male | 149 (33%) | 377 (57%) | 89 (36%) | 18 (24%) | 45 (28%) |
| Female | 299 (67%) | 282 (43%) | 158 (64%) | 58 (76%) | 117 (72%) |
| Age | | | | | |
| 50-59 | 211 (47%) | 172 (26%) | 101 (41%) | 38 (50%) | 21 (13%) |
| 60-69 | 131 (29%) | 204 (31%) | 85 (34%) | 19 (25%) | 42 (26%) |
| >69 | 106 (24%) | 283 (43%) | 61 (25%) | 19 (25%) | 99 (61%) |
| Disability | | | | | |
| Yes | 59 (13%) | 86 (13%) | 66 (27%) | 18 (24%) | 53 (33%) |
| No | 389 (87%) | 573 (87%) | 180 (73%) | 58 (76%) | 107 (66%) |
| Education | | | | | |
| No | 38 (8.5%) | 87 (13%) | 24 (9.7%) | 12 (16%) | 45 (28%) |
| Primary | 160 (36%) | 245 (37%) | 116 (47%) | 30 (39%) | 74 (46%) |
| Secondary | 152 (34%) | 189 (29%) | 77 (31%) | 23 (30%) | 27 (17%) |
| University | 98 (22%) | 136 (21%) | 30 (12%) | 11 (14%) | 16 (9.9%) |
| Fruits and Vegetables | | | | | |
| One per week or less | 37 (8.3%) | 61 (9.3%) | 24 (9.7%) | 7 (9.2%) | 16 (9.9%) |
| 4 times per week | 116 (26%) | 195 (30%) | 73 (30%) | 28 (37%) | 57 (35%) |
| One per day | 121 (27%) | 180 (27%) | 70 (28%) | 12 (16%) | 39 (24%) |
| Two or more per day | 174 (39%) | 223 (34%) | 80 (32%) | 29 (38%) | 50 (31%) |
| Physical Activity | | | | | |
| Never | 108 (24%) | 173 (26%) | 84 (34%) | 39 (51%) | 75 (46%) |
| One per month or less | 24 (5.4%) | 40 (6.1%) | 19 (7.7%) | 7 (9.2%) | 7 (4.3%) |
| Several times per week | 168 (38%) | 210 (32%) | 77 (31%) | 14 (18%) | 39 (24%) |

| | | | | | |
|---|---|---|---|---|---|
| All days | 148 (33%) | 236 (36%) | 67 (27%) | 16 (21%) | 41 (25%) |
| Alcohol Consumption | | | | | |
| Never | 172 (38%) | 217 (33%) | 110 (45%) | 50 (66%) | 99 (61%) |
| One per month or less | 28 (6.2%) | 38 (5.8%) | 19 (7.7%) | 6 (7.9%) | 15 (9.3%) |
| One per week or less | 108 (24%) | 143 (22%) | 54 (22%) | 11 (14%) | 23 (14%) |
| Several times per week | 140 (31%) | 261 (40%) | 64 (26%) | 9 (12%) | 25 (15%) |
| Smoke | | | | | |
| Neither smokes nor has smoked | 195 (44%) | 296 (45%) | 87 (35%) | 35 (46%) | 88 (54%) |
| Used to smoke | 175 (39%) | 265 (40%) | 112 (45%) | 22 (29%) | 57 (35%) |
| 1-10 cigarettes | 51 (11%) | 55 (8.3%) | 24 (9.7%) | 13 (17%) | 6 (3.7%) |
| >10 cigarettes | 27 (6.0%) | 43 (6.5%) | 24 (9.7%) | 6 (7.9%) | 11 (6.8%) |
| Job | | | | | |
| Active | 144 (32%) | 161 (24%) | 69 (28%) | 18 (24%) | 11 (6.8%) |
| Retiree | 158 (35%) | 355 (54%) | 90 (36%) | 29 (38%) | 98 (60%) |
| Unemployed | 54 (12%) | 49 (7.4%) | 28 (11%) | 7 (9.2%) | 6 (3.7%) |
| Domestic Work | 91 (20%) | 91 (14%) | 58 (23%) | 22 (29%) | 47 (29%) |
| Income | | | | | |
| <600€ | 30 (6.7%) | 41 (6.2%) | 18 (7.3%) | 17 (22%) | 16 (9.9%) |
| 601€-900€ | 73 (16%) | 122 (19%) | 47 (19%) | 15 (20%) | 52 (32%) |
| 901€-1200€ | 97 (22%) | 113 (17%) | 76 (31%) | 18 (24%) | 39 (24%) |
| 1201€-1800€ | 108 (24%) | 143 (22%) | 43 (17%) | 15 (20%) | 25 (15%) |
| >1800€ | 100 (22%) | 148 (22%) | 29 (12%) | 6 (7.9%) | 17 (10%) |
| No answer | 40 (8.9%) | 92 (14%) | 34 (14%) | 5 (6.6%) | 13 (8.0%) |
| COVID-19 | | | | | |
| Yes | 77 (17%) | 127 (19%) | 53 (21%) | 15 (20%) | 30 (19%) |
| No | 371 (83%) | 532 (81%) | 194 (79%) | 61 (80%) | 132 (81%) |
| COVID-19 Sequelae | | | | | |
| Yes | 17 (22%) | 21 (17%) | 12 (23%) | 6 (40%) | 8 (27%) |
| No | 60 (78%) | 106 (83%) | 41 (77%) | 9 (60%) | 22 (73%) |
| Primary Attention Visit | | | | | |
| Yes | 334 (75%) | 485 (74%) | 208 (84%) | 68 (89%) | 144 (89%) |
| No | 114 (25%) | 174 (26%) | 39 (16%) | 8 (11%) | 18 (11%) |
| Emergencies Visit | | | | | |
| Yes | 132 (29%) | 183 (28%) | 99 (40%) | 36 (47%) | 85 (52%) |
| No | 316 (71%) | 476 (72%) | 148 (60%) | 40 (53%) | 77 (48%) |
| Hospital Admission | | | | | |
| Yes | 35 (7.8%) | 60 (9.1%) | 30 (12%) | 10 (13%) | 35 (22%) |

| | | | | | |
|---|---|---|---|---|---|
| No | 413 (92%) | 599 (91%) | 217 (88%) | 66 (87%) | 127 (78%) |
| Specialist Visit | | | | | |
| Yes | 242 (54%) | 280 (42%) | 134 (54%) | 50 (66%) | 102 (63%) |
| No | 206 (46%) | 379 (58%) | 113 (46%) | 26 (34%) | 60 (37%) |

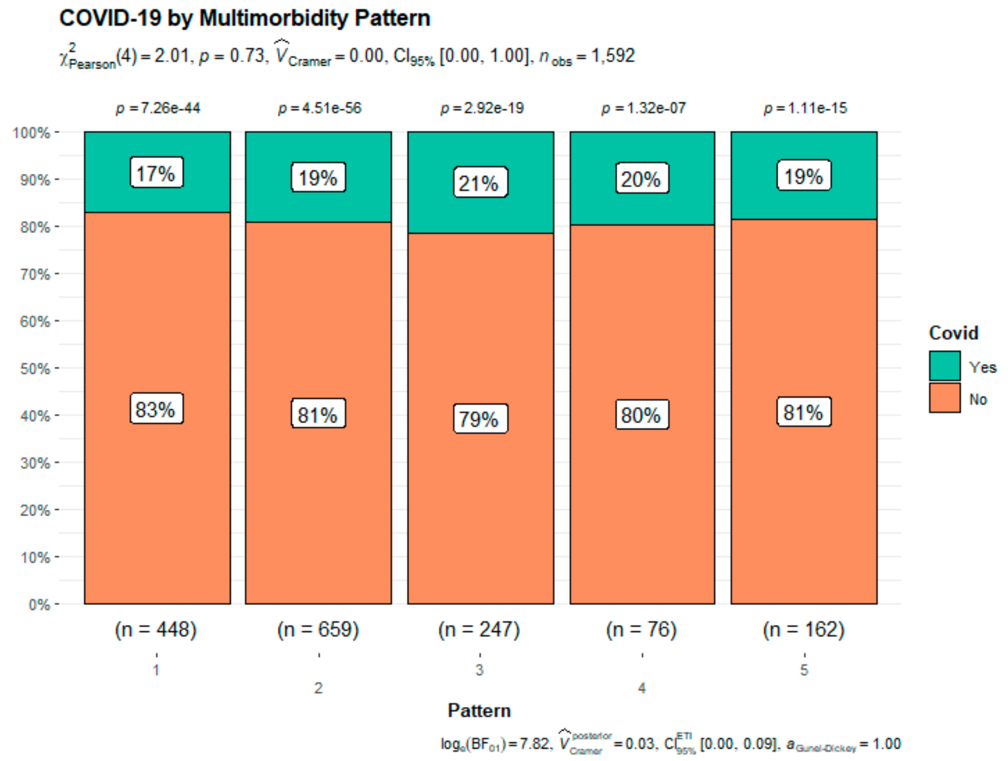


**Figure S1**: COVID-19 by Multimorbidity Pattern

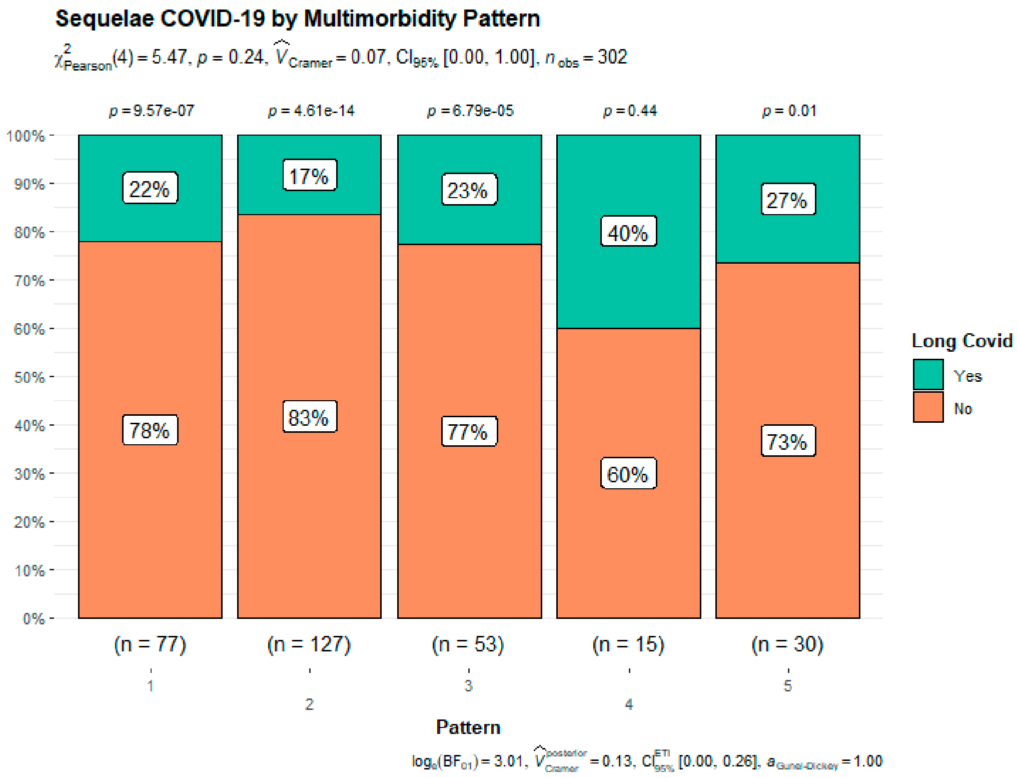


**Figure S2:** Sequelae COVID-19 by Multimorbidity Pattern
